# Supplementary material for: Body size information in large-scale acoustic bat databases
Source: PeerJ. 2018 Aug 24;6:e5370. doi: 10.7717/peerj.5370 (PMC6110253; doi:10.7717/peerj.5370)
Supplement: Supplemental Information 1 — A1: Detailed information on sampling design; A2:Habitat openness classification and relationship with the slope of the quasi-constant frequency; A3:Body mass, forearm length and sex in mist net dataset; A4:Analyses on geographic data subsets; A5:Influence of temperature measured during recording; A6:Influence of survey period and recording day in acoustic dataset; A7:Extended acknowledgements [file peerj-06-5370-s001.docx]

***Appendix A1 to A7: Supplementary information on methods and results***

**Body size information in large-scale acoustic bat databases**

Caterina Penone*, Christian Kerbiriou, Jean-François Julien, Julie Marmet, Isabelle Le Viol

*caterina.penone@gmail.com

**A1**: Detailed information on sampling design

**A2:** Habitat openness classification and relationship with the slope of the quasi-constant frequency

**A3:** Body mass, forearm length and sex in mist net dataset

**A4:** Analyses on geographic data subsets

**A5:** Influence of temperature measured during recording

**A6:** Influence of survey period and recording day in acoustic dataset

**A7:** Extended acknowledgements

**References**

**A1: Detailed information on sampling design**

In the French car-based bat monitoring (*Vigie-Chiro*), volunteers record along established circuits at night and at low speed (recording at constant speed: (25 ± 5 km/h)). The circuits exclude non paved roads, roads with high traffic and high-speed roads. All roads are of similar width, around 10 meters. The volunteers do the initial choice of the circuits because they can identify roads with the suitable characteristics from their knowledge of the region.

Observers were asked to choose a road circuit of at least 30 km and located within 10 km of their house. The circuit must not overlap itself. The second requirement is to design a circuit which crosses proportionally, as much as possible, the different habitats present in the area. The aim is to limit bias in habitat sampling (we assume that observers would tend to sample rich habitats). In order to generate randomization each circuit is then divided into ten 2-km road segments, separated by 1-km road segments without recording. Using this sampling design allowed us to obtain a good correlation between the proportion of each habitat sampled and the proportion of that habitat at the national scale (R² = 0.94). However, the urban areas were still slightly better represented than rural areas in our sample.

Records began 30 min after sunset during nights with temperatures greater than 12°C, wind speed less than 7km/h and without rain. We divided each 2-km road segment into 400-m sections using GIS (ARCGIS 9.3, ESRI). We allocated all echolocation calls detected along a segment to the coordinates of its central point using time and speed parameters.

*Bat recording characteristics*

Bat calls were detected using a Tranquility Transect Bat detector (Courtpan Design Ltd, UK) and recorded on a Zoom H2 digital recorder (Samson technologies, USA). All detectors were calibrated at the French National Museum History (MNHN) in order to obtain the same level of sensitivity. Therefore, sampling settings (sampling frequency and resolution) were the same in the whole geographic range. The signal from the condenser microphone of the bat detector was internally amplified and high pass filtered with a 5 kHz corner frequency. The Zoom H2 recorded the signal at 96 000 sample/s as PCM files. The information was stored in a Waveform Audio File Format (WAV), a format without compression.

*Bat species identification and sound parameters measurements*

We used Scan‘R (Binary Acoustic Technology, Tucson, Arizona) to automatically isolate each bat vocalization and measure a set of relevant parameters (Gannon et al. 2004; Obrist et al. 2004; Barataud 2012). The main relevant parameters measured by Scan’R that we used here, were: Call Duration (Dur), Highest (Fmax) and Lowest (Fmin) frequencies, Bandwidth (BW = Fmax – Fmin), Frequency at the strongest sound pressure level (Fdom), Characteristic frequency (LowFc), Mean Slope of the call, i.e., the average frequency modulation rate (Slope = BW/Dur), slope of the QCF part of the call (SlopeQCF). In France, other species with QFC between 41-52 kHz occur besides *P pipistrellus*, namely *P nathusii, P kuhlii, P pygmaeus* and *Miniopterus schreibersi*. Therefore, some errors in identification assignation might exist due to some overlap between acoustic repertories. However, we found similar proportions of these different species in both the acoustic and the capture datasets (Table A1.1), with *P. pipistrellus* being by far the most abundant species. The high difference in the relative abundance of *M. schreibersi* in the two datasets is due to the fact that some captures may be performed close to roosts for specific studies, while the acoustic survey sampling is designed to be representative of the main habitats.

The French national bat-monitoring program has a validation system where the results of automatic identifications are checked by experts. The results of the cross-validation on more than 58% of the data indicate that for 8% of the calls identified as *P. kuhlii* it was not possible to refute potential contact of *P. nathusius* and among the *P. pipistrellus* less than 0.1% could be *P. nathusii*.

**Table A1.1** Proportion of species (%) with potentially a QCF frequencies between 41-52 kHz according to the dataset

|  | **Acoustic dataset** |  | **Capture dataset** |
| --- | --- | --- | --- |
| *Miniopterus schreibersi* | 0.02 |  | 7.74 |
| *Pipistrellus kulhii* | 12.20 |  | 6.07 |
| *Pipistrellus nathusius* | 1.19 |  | 3.40 |
| *Pipistrellus pipistrellus* | 85.97 |  | 81.24 |
| *Pipistrellus pygmaeus* | 0.62 |  | 1.56 |

**A2: Habitat openness classification and relationship with the slope of the quasi-constant frequency (SlopeQCF)**

In the French national bat monitoring, volunteers were also involved in collecting detailed habitat variables (Kerbiriou et al. 2010). Observers described the habitat every 400 meters, i.e., five times per 2-km segment. Each circuit was thus associated with 50 habitat descriptions. Observers classified habitats within a range of 100 meters around the road. The appropriate habitat codes are chosen from an established hierarchical system which is very similar to a widely used habitat code in bird monitoring schemes (see Crick (1992) for the UK and Barnagaud et al. (2012) for the French monitoring), but adapted to account for bat foraging specificities and particularly linear elements of the landscape (for more details about the French Bat monitoring see the website [http://vigienature.mnhn.fr/page/releves-d-habitats]).

In the present work, in order to have a rough measure of habitat openness, we simplified this classification into two main categories, as follows:

- open habitat (2406 segments): included arable land, permanent crops, pastures, shrubs, lawns, urban spaces, rocks and water bodies

- cluttered (i.e. habitats with closed and/or dense vegetation, 1389 segments): included hedges and all types of dense young and old forests)

Habitat openness has been shown to have an effect on both the frequency and the shape of bat calls, including for *P. pipistrellus* (Kalko and Schnitzler 1993). Bats emit higher frequency calls with a steeper shape (thus high values of the slope of the quasi-constant frequency, SlopeQCF) when they fly in cluttered habitats (habitats with closed and dense vegetation). When they fly in open habitats, their calls harbour shallower (corresponding to low values of SlopeQCF) and lower frequencies (Figure A2.1). We thus tested the relationship between the SlopeQCF and habitat openness using a t-test. We also tested whether characteristic frequency (LowFc) differed between open and cluttered habitats using a t-test. We found that the SlopeQCF was significantly higher in cluttered habitats compared to open habitats: t = -4.0803, df = 2741.176, p-value = 4.626e-05. This was consistent with previous studies (Schnitzler and Kalko 2001). The characteristic frequency in cluttered habitats was 46.8±1.9 and 46.9±1.9 in open habitats (t= -2.33, df=2861, pvalue=0.02).

Because we found that SlopeQCF was strongly related to a simplified measure of habitat openness and in order to avoid multicollinearity by adding both habitat and SlopeQCF into the models, we chose to add only SlopeQCF into our models.

**Figure A2.1**: Example of calls with high (A) and low (B) values of SlopeQCF (slope of the quasi-constant frequency). Fc (or LowFc) is the frequency of the end of the quasi-constant frequency part of the signal and was used for species identification.

**A3: Body mass, forearm length and sex in mist net capture data**

### We assessed the relationship between the sex of the captured individuals and their body mass and forearm length. As expected we found a strong link between sex, body mass and forearm length (Fig. A3.1): females were larger and heavier than males.

| 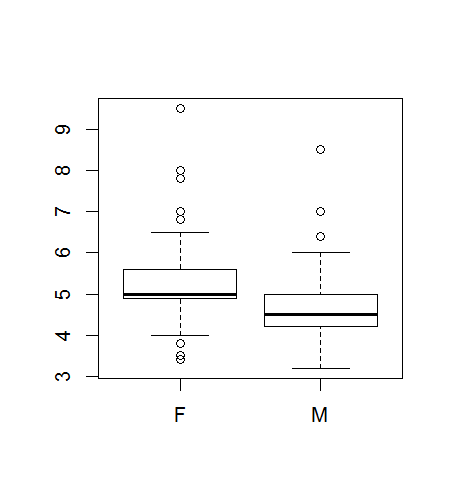  Forearm length | 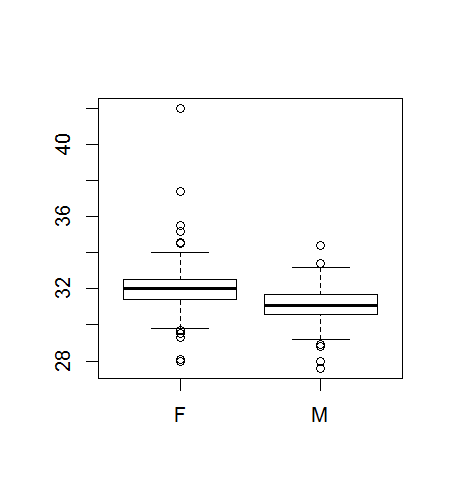  Body mass |
| --- | --- |

Figure A3.1: Variation of forearm length and body mass according to sex (F: female, M: male)

We also found that forearm length of *P. pipistrellus* was proportional to the mass of the individuals by a 0.29 ratio a value, close to that found by Norberg (1981) for Vespertilionids. This relationship was robust for both females (Fig. A3.2-A) and males (Fig. A3.2-B) (both *Pvalues* < 0.0001), and was maintained even when sex effect was not taken into account (*Pvalue* < 0.0001).

| 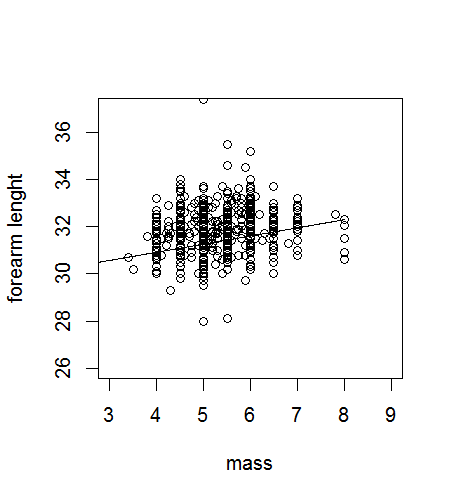  Forearm length  Body mass  **A** | 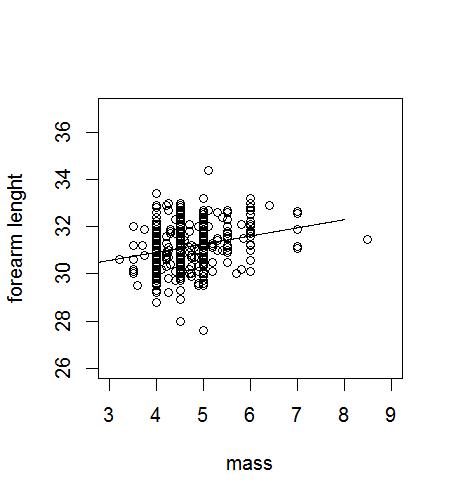  Body mass  Forearm length  **B** |
| --- | --- |

Figure A3.2: Relationship between forearm length and body mass for females (A) and males (B) of *P. pipistrellus*

**A4: Analyses on geographic data subsets**


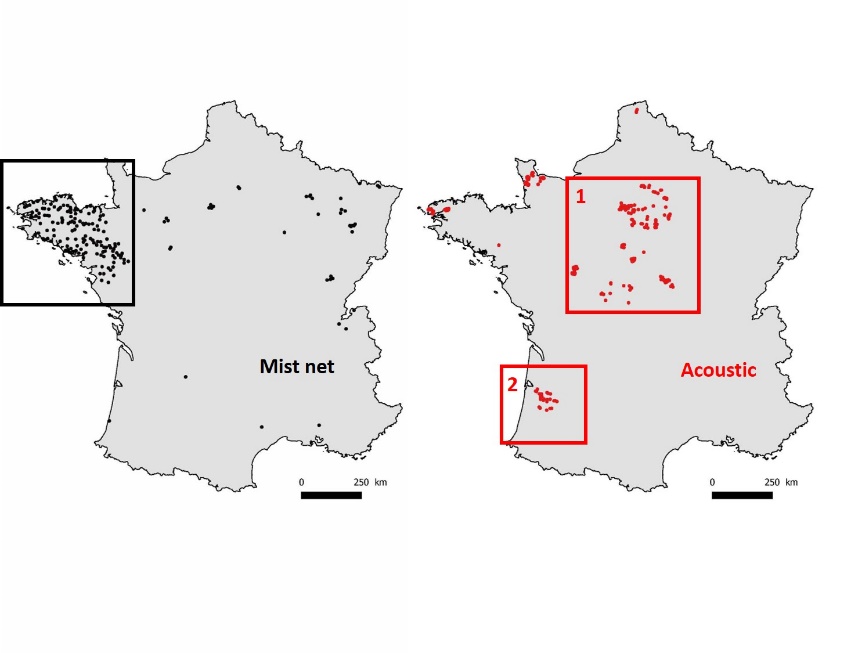


**Figure A4.1**: Map of France with locations where bats were captured (mist net dataset, black points) and recorded (acoustic dataset, red points), and delimitations of subsets for analyses. The black square indicates the area with high concentration of points for the mist net dataset (northwestern subset). The red square 1 indicates the area with high concentration of points for the capture dataset (central northern subset). The red square 2 indicates southern points used for sensitivity analysis for potential species identification issues in the acoustic dataset (southern subset).

*Mist net dataset*

Our mist net capture dataset was not evenly distributed in space, with a high concentration of data points in the western part of France and a weak latitudinal representation. In order to check if our results were not biased by data sampling, we reproduced the same analysis described in the manuscript on a data subset including only high point densities (i.e. Northwestern France, Fig.A4.1). We found very similar results ad for the whole dataset (Table A4.1).

**Table A4.1:** Results of the linear mixed-effects models on a subset of the mist net dataset including only the north-western dense data-point areas, see Figure A4.1 (response variable: forearm length).

M.PC1 and M.PC2 are the principal components of a PCA including climatic and geographic (latitude, longitude) variables. The estimate value for Males is relative to the estimate value of the Females.

*Acoustic dataset*

Similarly to the previous analysis (on the mist net dataset), we reproduced the same analysis described in the manuscript on a data subset including only high point densities (i.e. central northern France). We found very similar results ad for the whole dataset (Table A4.1). We found the same results as for the whole dataset. The PC2, representing latitude and temperature, was positively correlated to the characteristic frequency, meaning that larger individuals (lower frequencies) were recorded in places characterized by low temperatures and located in northern latitudes (Table A4.2).

**Table A4.2:** Results of the linear mixed-effects models on a subset of the acoustic dataset including only the dense data-point areas, see Figure A4.1 (response variable: characteristic frequency). A.PC1 and A.PC2 are the principal components of a PCA including climatic and geographic (latitude, longitude) variables.

Sound species identification was done by trained volunteers, and verified by the French national bat monitoring coordinators (Jean-François Julien and Christian Kerbiriou), the risk of confusing species is hence very low. However to be highly cautious and take into account the fact that some signals may have been attributed to *Pipistrellus pipistrellus* but, in fact emitted by *Pipistrellus pygmaeus* (which mostly occurs in the South of France, is smaller and emits higher frequency signals (Mayer and Von Helversen 2001)), we repeated our analyses separately for northern and southern France (Figure A4.1). We fitted the same model described in the method section of the manuscript. We found that the PC2 did not show any significant relationship with the characteristic frequency. However, the PC1, representing longitude, temperature variability and precipitations, was negatively correlated to characteristic frequency, meaning that larger individuals (lower frequencies) were recorded in places characterized by high precipitation, low temperature range and located in western France (Table A4.2). This was consistent with the results found using the mist net capture dataset. In the southern part of the dataset the data points are much more oriented along an east-west gradient (similarly to the mist net dataset), therefore this result might be expected and confirms the validity of sound data to capture body-size variation in space.

**Table A4.3:** Results of the linear mixed-effects models on a subset of the acoustic dataset including only the southern circuits, see Figure A4.1 (response variable: characteristic frequency). A.PC1 and A.PC2 are the principal components of a PCA including climatic and geographic (latitude, longitude) variables.

**A5: Influence of temperature measured during recording**

The local temperature during recording might have an effect on call detection. Because it is a parameter measured by the observers, we had information for only 44 circuits over 76. In order to assess the importance of this variable and its potential bias on our results, we ran the analyses on a subset including only these 44 circuits.

We fitted the lme (linear mixed-effects models) with and without the local temperature; we then compared the AIC of the two models and the significance of all the other variables.

The AIC of the model was slightly lower when the local temperature was included in the model. The results thus suggest that this variable might have a small importance on acoustic detection of *P. pipistrellus*. However, the relationship between the characteristic frequency and the principal components representing geography and climate remained unchanged when this variable was included in the model. Therefore not including it in the analyses did not seem to bias our result. However it highlights the importance of measuring it in future acoustic monitoring.

**Table A5.1:** Results of the linear mixed-effects models using acoustic data without the local temperature measured during recording (model 1) and with this variable (model 2) (response variable: characteristic frequency). A.PC1 and A.PC2 are the principal components of a PCA including climatic and geographic (latitude, longitude) variables.

**A6: Influence of survey period and recording day in acoustic dataset**

The day of the year in which the bats were recorded could potentially be more informative than the survey period, but we had this information for only 45% of the data points. We thus tested the effect of the day of recording on this data subset for the first survey. The results were similar in both models (Table A6.1).

**Table A6.1:** Results of the linear mixed-effects models using acoustic data, including the day of the year in which the bats were recorded. The first model (top, n=994) includes data for the first survey only, the second (bottom, n=2164) includes all data with available information on the day of recording. A.PC1 and A.PC2 are the principal components of a PCA including climatic and geographic (latitude, longitude) variables.

**A7: Extended acknowledgments - Volunteers involved in collecting mist net capture and acoustic data**

We deeply thank all people that collected mist net and acoustic data and through which the achievement of this paper has been made possible.

We thank batworkers who provided mist net data for *P.pipstrellus*: Office National des Forêts (Réseau Mammifères), Groupe Mammalogique Breton, Groupe Chiroptères Corse, CPEPESC Lorraine, Jocelyn Fonderflick, David Sarrey, Maxime Leuchtmann and Benjamin Allegrini.

We also thank all volunteers who took part in the French Bat Monitoring Programme, a large-scale survey which relies entirely on them: Alain Abba, Daoudou Ahamada, Olivier Allenou, Quentin Amand, Karine Ancrenaz, Ronan Arhuro, Edith Armange, Jacqueline Armange, Arnaud Bak, Yves Bas, Franz Barth, Jean- Pierre Bavent, Paul Ballongue, Joël Bec, Isabelle Bellier,Yannick Bernicot, Elodie Bideau, Romain Bion, Quentin Boisbouvier, Joisselin Boireau, Laurent Bokhor, Yves Bolnot ,Sandrine Bouligand, Annick Boutard, Pierre Boyer, Sandrine Bracco, Célia Bresson, Alexane Broussin, Matthieu Buis, Nathalie Carnino, Bruno Cart, Livio Casella, Pascal Cavalin, Julien Cavallo, Annie Chapalain, Claude Chapalain, Frédéric Chapalain, Thomas Chatton, Dominique Chavy, Nicolas Chenaval, Pierre Chico-Sarro, Manuel Chrétienne, Yann Coray, Julie Coulhon, Denis Couvet, Flore Cambon, François Collin, Cindy Da Costa, Hervé Dallemagne, Thomas Darnis, Yves David, Leïla Debiesse, Louis De Redon, Guy Derivaz, Philippe Defernez, Robin Dérozier, Nicolas Deguines, Emilie Desrousseaux Michel Di Maggio, Romuald Dohogne, Samuel Dorange, Chloé Dordonnat, Aggeleki Doxa, Thomas Dubos, Grégory Duclaud, Léa Dufrêne, Guillaume Duthion, Cécile Edelist, Alexandre Emerit, Gilles Faggio, Anne Ferment, Samuel Ferreira, Grégory Fiquet, Cyrille Frey, Nicolas Fillol, Ondine Filipi- Codaccioni, Nicolas Flament, Benoît Fontaine, Céline Fontaine, Jérôme Fouert-Pouret, Marie-Hélène Froger, Cécile Hignard, Anne-Sophie Gadot, Laurent Gager, Yann Gager, Miguel Gailledrat, Gilles Galbrun, Sébastien Galtier, Sébastien Gautier, Amandine Gasc, Loic Gosselin, Cédric Gouxette, Gérard Goujon, Anne-Laure Gourmand, Antoine Griboval, Charlène Guillon, Jean Guinard, Benjamin Guyonnet, Mélanie Hinz, Alexandre Haquart, Clément Heroguel, Bruno Honoré, Colette Huot-Daubremont, William Huin, Emmanuel Jacob, Frédéric Jiguet, Florie Johanot, Jean-François Julien, Christian Kerbiriou, Roman Landouzy, Sabrina Languin, Marion Laprun, Cécile Larivière, Marine Lauer, Thomas Le Campion, Benoit Lecaplain, Mélissa Leculier, Jean-Baptiste Leculier, Frédéric Leculier, Virginie Ledez, Kévin Lelarge, Pascal Lemoine, Lénaïg Lenen, Mathilde Lesur, Sylvain Letourneau, Maryse Legendre, Philippe Legendre, Arnaud Le Saout, Frédéric Levé, Isabelle Le Viol, Catherine Longuet, Romain Lorrillière, Jean Pierre Lair, Eléonore Lefebvre, Laura Lugris, Jean Marc Lustrat, Nathalie Machon, Olivia Magnoux, Sylvain Mahuzier, Louis Manche, Julie Maratrat, Julie Marmet, Alexandre Mari, Olivier Matton, Jean-François Magne, Charlie Mangin, François Martin, Julien Masquelier, Adams Mayer, Antoine Meirland, Sébastien Merle, Laurent Mercier, Anne Métaireau, Robert Meunier, Elouan Meyniel, Jean-Batiste Mihoud, Didier Montfort, Sylvain Montagner, Justine Mougnot, Jean Pierre Moussu, Romain Morvan, Bruno Munilla, Dominique Munilla, Gilles Naudet, Ronan Nédelec, Nadine Nicolas, Issa Nidal , Grégory Patek, Christophe Parisot, Roman Pavisse, Marie Pellé, M. Penpeny, Stéphane Petit, Hérvé Picq, Yannick Pochon, Arthur Pommerais, Emmanuelle Porcher, Thomas Radigois, Olivier Renault, Sylvain Richier, Jean marc Riouallen, Gaétan Rizet, Loïc Robert, Magali Roche , Dominique Rombaut, Philippe Roy, Loic Salaun, Etienne Sarrazin, David Sautet, Maurevas Sempé, Geoffrey Stevens, Anthony Sturbois, Silva Régina, Jean-Marc Riou, Franck Simmonnet, Nathalie Sionneau, Bérengère Soye, Anne Thebault, Anne Tessedre, Celine Teplitsky, Vincent Ternois, Marion Touchard, Jean-Paul Urcun, Denis Vandromme, Pauline Van Laere, Fabien Verfaille, Alan Vergnes, Marylène Vergnol, Marie-Laure Villa , Sophie Wrobel, Maxime Zucca.

**References**

Barataud M (2012) Écologie acoustique des chiroptères d’Europe: identification des espèces, étude de leurs habitats et comportements de chasse. Biotope and Muséum National d’Histoire Naturelle, Mèze & Paris, France

Barnagaud J-Y, Devictor V, Jiguet F, et al (2012) Relating habitat and climatic niches in birds. PLoS One 7:e32819.

Crick HQP (1992) A bird-habitat coding system for use in Britain and Ireland incorporating aspects of land-management and human activity. Bird Study 39:1–12. doi: 10.1080/00063659209477092

Gannon WL, O’Farrell MJ, Corben C, Bedrick EJ (2004) Call character lexicon and analysis of field recorded bat echolocation calls. In: Echolocation in bats and dolphins. University of Chicago Press, Chicago, IL, p 604

Kalko EV, Schnitzler H-U (1993) Plasticity in echolocation signals of European pipistrelle bats in search flight: implications for habitat use and prey detection. Behav Ecol Sociobiol 33:415–428. doi: 10.1007/BF00170257

Kerbiriou C, Bas Y, Dufrêne L, et al (2010) Long term trends monitoring of bats, from biodiversity indicator production to species specialization assessment. In: Society for Conservation Biology. 24th Annual Meeting. Edmonton, Alberta, Canada,

Mayer F, Von Helversen O (2001) Sympatric distribution of two cryptic bat species across Europe. Biol J Linn Soc 74:365–374. doi: 10.1111/j.1095-8312.2001.tb01398.x

Norberg U (1981) Allometry of bat wings and legs and comparison with bird wings. Philos Trans R Soc B Biol Sci 292:359–398.

Obrist MK, Boesch R, Flückiger PF (2004) Variability in echolocation call design of 26 Swiss bat species: consequences, limits and options for automated field identification with a synergetic pattern recognition approach. Mammalia 68:307–322.

Schnitzler H-U, Kalko EK V. (2001) Echolocation by insect-eating bats. Bioscience 51:557–569. doi: 10.1641/0006-3568(2001)051[0557:EBIEB]2.0.CO;2
